# Trial design and rationale for APOLLO, a Phase 3, placebo-controlled study of patisiran in patients with hereditary ATTR amyloidosis with polyneuropathy

**DOI:** 10.1186/s12883-017-0948-5

**Published:** 2017-09-11

**Authors:** David Adams, Ole B. Suhr, Peter J. Dyck, William J. Litchy, Raina G. Leahy, Jihong Chen, Jared Gollob, Teresa Coelho

**Affiliations:** 1 CHU Hôpital Bicêtre, Le Kremlin-Bicêtre CEDEX, Paris, France; 2 Department of Public Health and Clinical Medicine, Umeå University Hospital, Umeå, Sweden; 3 Department of Neurology, Mayo Clinic, Rochester, MN USA; 4 Alnylam Pharmaceuticals, Cambridge, MA USA; 5 Hospital Santo António, Centro Hospitalar do Porto, Porto, Portugal

**Keywords:** Patisiran, APOLLO, RNA interference, hATTR amyloidosis, mNIS+7, Methods, Polyneuropathy

## Abstract

**Background:**

Patisiran is an investigational RNA interference (RNAi) therapeutic in development for the treatment of hereditary ATTR (hATTR) amyloidosis, a progressive disease associated with significant disability, morbidity, and mortality.

**Methods:**

Here we describe the rationale and design of the Phase 3 APOLLO study, a randomized, double-blind, placebo-controlled, global study to evaluate the efficacy and safety of patisiran in patients with hATTR amyloidosis with polyneuropathy. Eligible patients are 18–85 years old with hATTR amyloidosis, investigator-estimated survival of ≥2 years, Neuropathy Impairment Score (NIS) of 5–130, and polyneuropathy disability score ≤IIIb. Patients are randomized 2:1 to receive either intravenous patisiran 0.3 mg/kg or placebo once every 3 weeks. The primary objective is to determine the efficacy of patisiran at 18 months based on the difference in the change in modified NIS+7 (a composite measure of motor strength, sensation, reflexes, nerve conduction, and autonomic function) between the patisiran and placebo groups. Secondary objectives are to evaluate the effect of patisiran on Norfolk-Diabetic Neuropathy quality of life questionnaire score, nutritional status (as evaluated by modified body mass index), motor function (as measured by NIS-weakness and timed 10-m walk test), and autonomic symptoms (as measured by the Composite Autonomic Symptom Score-31 questionnaire). Exploratory objectives include assessment of cardiac function and pathologic evaluation to assess nerve fiber innervation and amyloid burden. Safety of patisiran will be assessed throughout the study.

**Discussion:**

APOLLO represents the largest randomized, Phase 3 study to date in patients with hATTR amyloidosis, with endpoints that capture the multisystemic nature of this disease.

**Trial registration:**

This trial is registered at clinicaltrials.gov (NCT01960348); October 9, 2013.

## Background

Hereditary ATTR (hATTR) amyloidosis, formerly known as familial amyloid polyneuropathy (FAP), is a progressive, life-threatening disease caused by misfolded transthyretin (TTR) protein that accumulates as amyloid fibrils in multiple organs, including the nerves, heart, and gastrointestinal tract [1, 2]. hATTR amyloidosis is a multisystemic disease with a heterogeneous clinical presentation, including sensory, motor, autonomic, and cardiac symptoms that are often concurrent [3–6]. The unrelenting disease course begins with unimpaired ambulation (FAP stage 1 [7]), then requirement for assistance with ambulation (FAP stage 2), which proceeds to wheelchair confinement (FAP stage 3), with patients experiencing a range of life-impacting symptoms that include burning neuropathic pain, loss of sensation in hands and feet, diarrhea/constipation, sexual impotence, and dizziness/fainting [8–10]. The median survival for patients with hATTR amyloidosis with polyneuropathy is reported as 5–15 years from diagnosis [9, 11–13].

To date >120 *TTR* mutations have been reported [14]; some mutations are more strongly associated with polyneuropathy (e.g. V30M [8]), and others with cardiomyopathy (e.g. V122I [15]). However, this genotype–phenotype association likely represents an over-simplification, with wide variation in presentation reported between genotypes and a mixed phenotype commonly observed [3, 16].

Effectively quantifying the disease burden in hATTR amyloidosis remains challenging, as there is no single test that captures the constellation of symptoms and the multisystemic nature of the condition. Indeed, even assessment of all signs and symptoms of polyneuropathy requires numerous measures, which recent data suggest are not adequately captured in current tests [17]. For example, the Neuropathy Impairment Score (NIS)-Lower Limb (NIS-LL) is based on examination of lower limbs only, so dysfunction occurring in other areas as the disease progresses cannot be captured. The widely used NIS overcomes this limitation through clinical examination of lower limbs, upper limbs, and cranial nerves, although this tool does not include nerve conduction scores, which are critical to assess the axonal neuropathy which progresses during the disease course [6, 8, 12]. In addition, the NIS does not adequately address sensory loss over the body, which is a hallmark of the disease [18]. Ultimately, scoring the full range of neuropathic impairment likely requires a combined measure of the type, severity, and distribution of neurologic signs and symptoms [17]. There is also a limited set of tools that have demonstrated utility in assessment of quality of life (QoL) and physical functioning in hATTR amyloidosis, primarily the Norfolk Quality of Life-Diabetic Neuropathy (QOL-DN), EuroQoL 5-Dimensions (EQ-5D™), and Short-Form 36 (SF-36) Health Survey questionnaires [19–24]. Consequently, the need remains for clinical trials that include a comprehensive set of measures to investigate disease progression and the effects of treatment on patient well-being and function.

As TTR is produced predominantly in the liver [25, 26] orthotopic liver transplantation (OLT) is a well-recognized and effective treatment strategy for replacing mutant TTR protein [27, 28]. However, OLT is only recommended for patients with early-stage hATTR amyloidosis, and survival varies according to modified body mass index (mBMI), disease duration/severity, and *TTR* mutation [8, 28, 29]. The procedure itself is also limited by issues such as cost, donor availability, cardiac involvement, and toxicities associated with immunosuppression [2, 30]. Furthermore, disease progression can continue after OLT as a result of amyloid fibril deposition from wild-type TTR [31, 32]. More recently, the pharmacotherapies tafamidis and diflunisal, which stabilize the TTR complex and prevent protein misfolding (TTR tetramer stabilizers), have been utilized. Tafamidis was approved in Europe for patients with hATTR amyloidosis with early-stage neurologic disease (FAP stage 1), despite limited data, with authorization under “exceptional circumstances” due to the clinical unmet need [33]. Subsequent approvals for treatment of symptomatic polyneuropathy have followed in regions of Latin America and Asia. Diflunisal, a non-steroidal anti-inflammatory drug, has been used off label for management of hATTR amyloidosis [34]. In clinical studies, both compounds have slowed progression of neurologic impairment and were generally well tolerated [19, 24, 35]. However, progression of neuropathy symptoms or disability is still observed in some patients [24, 35–37], and tafamidis may have reduced efficacy in patients with more severe disease [38]. Thus, there remains a need for novel treatment options for hATTR amyloidosis.

Patisiran is an investigational RNA interference (RNAi) therapeutic in clinical development for hATTR amyloidosis. Patisiran is a small interfering RNA (siRNA) that targets a sequence of mRNA conserved across wild-type and all TTR variants and can thereby reduce serum levels of both wild-type and pathogenic (mutated) protein [39]. It is formulated as lipid nanoparticles which direct it to the liver, the primary source of circulating TTR [39]. The benefits of lowering mutant TTR levels in patients with hATTR amyloidosis have been demonstrated by OLT [40, 41], and data from other amyloidoses show that clinical outcomes can be improved by reducing amyloidogenic protein [42–44]. In a Phase 2 study (NCT01617967) of 29 patients with hATTR amyloidosis, two doses of patisiran 0.3 mg/kg every 3 weeks reduced mean serum TTR levels by approximately 80% [45]. This potent TTR knockdown was observed for both wild-type and mutant (V30M) forms of TTR, and in patients who were concurrently receiving TTR tetramer stabilizers [45]. An ongoing open-label extension study (NCT01961921) has provided encouraging data, with stable measures of neuropathy impairment and patisiran being generally well tolerated through 24 months of treatment [46].

Collectively, these data support the hypothesis that TTR reduction has the potential to stabilize the progression of – or even reverse – neuropathy in patients with hATTR amyloidosis. To date, APOLLO is the largest Phase 3 clinical study in patients with hATTR amyloidosis, designed to assess the safety and efficacy of patisiran on neurologic function and QoL.

## Methods

### Study oversight

This study is conducted according to the guidelines of the International Conference on Harmonisation, the World Health Organization’s Declaration of Helsinki, and the Health Insurance Portability and Accountability Act of 1996. Written informed consent is obtained from all patients who participate in the study, prior to assessment of eligibility. The study protocol was approved by the local Institutional Review Boards and Ethics Committees, and all subsequent protocol amendments underwent the same approval procedure. A clinical monitor, as a representative of Alnylam, will follow the study through periodic site visits and frequent telephone/written contact. A Data Monitoring Committee will be implemented for the study and provide independent evaluation to ensure patient safety. This trial is registered at www.clinicaltrials.gov (NCT01960348).

### Study overview and setting

**Fig. 1:**
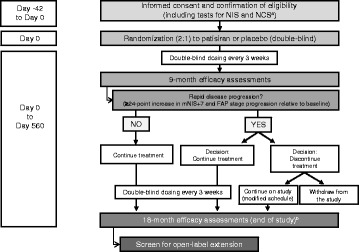
APOLLO study schematic. ^a^Karnofsky performance status, New York Heart Association class, safety, and TTR genotyping also assessed as part of eligibility criteria. ^b^18-month efficacy assessments: mNIS+7, NIS+7, FAP score, polyneuropathy disability stage, nerve fiber density, dermal amyloid burden, modified body mass index, timed 10-m walk test, grip strength test, Composite Autonomic Symptom Score-31 questionnaire, Norfolk Quality of Life-Diabetic Neuropathy questionnaire, EuroQol 5-Dimensions questionnaire, Rasch-built Overall Disability Scale, echocardiogram, and cardiac biomarkers. *FAP* familial amyloidotic polyneuropathy; *IV* intravenous; *mNIS+7* modified NIS+7; *NCS* nerve conduction study; *NIS* Neuropathy Impairment Score

APOLLO is a randomized, multicenter, international, double-blind, placebo-controlled, Phase 3 study designed to evaluate the efficacy of patisiran and establish the safety of chronic dosing in adult patients with symptomatic hATTR amyloidosis with polyneuropathy. A study schematic is shown in Fig. 1. Patients are recruited from 46 sites across 19 countries (United States, France, Taiwan, Spain, Japan, Germany, Mexico, Portugal, South Korea, Sweden, Bulgaria, Italy, Canada, Turkey, Cyprus, Brazil, Netherlands, United Kingdom, and Argentina). All but two of the sites are academic hospitals.

### Eligibility

At screening, patients are 18–85 years old, with a diagnosis of hATTR amyloidosis, a documented *TTR* mutation, and investigator-estimated survival of ≥2 years. Eligible patients have an NIS of 5–130, polyneuropathy disability (PND) score ≤IIIb, and adequate biochemical liver function. The NIS range of 5–130 was selected to include patients with disease sufficiently advanced to show progression in the placebo group, but not so advanced as to preclude detection of a change in disease status. Patients with previous OLT or sensorimotor/autonomic neuropathy due to other known causes are not included in the study. The key inclusion and exclusion criteria are summarized in Table 1.

**Table 1:** Key inclusion and exclusion criteria for the APOLLO study

Inclusion criteria	Exclusion criteria
• Diagnosis of hATTR amyloidosis with documented mutation • Anticipated survival ≥2 years • Aged 18–85 years (inclusive) • NIS of 5–130 • PND score ≤IIIb • NCS sum of the sural SNAP, tibial CMAP, ulnar SNAP, ulnar CMAP, and peroneal CMAP of ≥2 points • Karnofsky performance status ≥60% • Absolute neutrophil count ≥1500 cells/mm^3^ • Platelet count ≥50,000 cells/mm^3^ • Adequate biochemical liver function^a^ • Serum creatinine ≤2 × ULN	• Previous liver transplantation, or liver transplantation planned during study period • Sensorimotor or autonomic neuropathy not related to hATTR amyloidosis • Primary or leptomeningeal amyloidosis • Type 1 diabetes • Type 2 diabetes for ≥5 years • Active hepatitis B or C, or HIV infection • NYHA heart failure classification >2 • Acute coronary syndrome within past 3 months • Uncontrolled cardiac arrhythmia or unstable angina • Severe reaction to a liposomal product or hypersensitivity to oligonucleotides • Unable to take premedications • Received an investigational agent within 30 days/5 half-lives (whichever is longer) of study drug administration • Currently taking tafamidis, diflunisal, doxycycline, or tauroursodeoxycholic acid

*CMAP* compound muscle action potential; *hATTR amyloidosis* hereditary transthyretin-mediated amyloidosis; *NCS* nerve conduction study; *NIS* Neuropathy Impairment Score; *NYHA* New York Heart Association; *PND* polyneuropathy disability; *SNAP* sensory nerve action potential; *ULN* upper limit of normal

^a^Aspartate transaminase and alanine transaminase levels ≤2.5 × ULN; total bilirubin levels within normal limits; international normalized ratio ≤2.0

### Study design

Patients are randomized to receive either patisiran 0.3 mg/kg or placebo (normal saline 0.9%) once every 3 weeks for 18 months. Study drug is administered as an intravenous infusion over 70 min (1 mL/min for the first 15 min, then 3 mL/min thereafter).

Patients have the option of discontinuing study drug if they experience a protocol-defined rapid disease progression at 9 months, defined as ≥24-point increase in mNIS+7 from baseline (estimated 24-point increase in mNIS+7 score in the placebo group expected by 18 months based on natural history study [47]), and FAP stage [7] progression relative to baseline, confirmed by an external adjudication committee. Such patients may receive alternative therapy (local standard of care) and will be monitored based on a modified visit schedule if they decide to stay on study. All patients who complete the final 18-month assessment are eligible to screen for an open-label extension study of long-term patisiran treatment (NCT02510261).

### Premedication

To reduce the likelihood of infusion-related reactions, patients are to receive the following premedications or equivalent at least 60 min before each study drug infusion: dexamethasone; oral acetaminophen/paracetamol; an H_2_ blocker (e.g., ranitidine or famotidine); and an H_1_ blocker (e.g., diphenhydramine).

### Concomitant medications

The use of tafamidis, diflunisal, doxycycline, tauroursodeoxycholic acid, or any investigational agent other than patisiran is prohibited during study participation. These agents may have been used before screening, but a wash-out period is mandated (14 days for tafamidis, doxycycline, or tauroursodeoxycholic acid; 3 days for diflunisal). Palliative and supportive-care medications are permitted during the study.

### Stratification, randomization, and blinding

Patients are randomized in a 2:1 (patisiran:placebo) ratio using an interactive response system. Treatment arms are balanced at study entry for: NIS (5–49 vs 50–130), early-onset V30M disease (age < 50 years at onset) vs all other mutations (including late-onset V30M), and previous use of tafamidis or diflunisal. Patients and study personnel who monitor patients during infusions and perform clinical assessments are blinded to the study treatment. Unblinded personnel and pharmacists prepare the drug for administration, but are not involved in patient management or safety or efficacy assessments. Details of patients who discontinue study drug at 9 months due to rapid disease progression remain blinded throughout the study.

### Efficacy assessments

#### Primary outcome measure: mNIS+7

The primary objective is to determine the difference between the patisiran and placebo groups in change from baseline in mNIS+7 at 18 months (Table 2). The mNIS+7 used in APOLLO has been modified from the original NIS+7 to better characterize and quantify sensation all over the body, autonomic function, and nerve conduction changes associated with hATTR amyloidosis progression [17]. A summary of the scoring components of the NIS+7 and mNIS+7 is provided in Table 3.

**Table 2:** Study objectives

Objective	
Primary	• Determine the efficacy of patisiran by evaluating the difference between the patisiran and placebo groups in the change from baseline of mNIS+7 at 18 months
Secondary (hierarchical ordering)	• Norfolk Quality of Life-Diabetic Neuropathy questionnaire • NIS-weakness score • Level of disability (Rasch-built Overall Disability Scale) • Timed 10-m walk test • mBMI^a^ • Composite Autonomic Symptom Score questionnaire
Exploratory	• NIS + 7 score • Grip strength • EuroQol 5-Dimensions questionnaire (EQ-5D-5L index, EQ VAS) • Large versus small nerve fiber function^b^ • Sensory and autonomic innervation and analysis of nerve fiber density and sweat gland nerve fiber density • Dermal amyloid content on skin biopsy • FAP stage and PND score • Cardiac measures (echocardiogram, troponin I, and NT-proBNP levels) • Pharmacodynamic biomarkers (TTR, RBP, and vitamin A) • Proportion of patients with rapid disease progression at 9 months • Lower limb nerve injury via voluntary magnetic resonance neurography • Columbia-Suicide Severity Rating Scale (C-SSRS)

*FAP* familial amyloidotic polyneuropathy; *mBMI* modified body mass index; *mNIS* modified Neuropathy Impairment Score; *NIS* Neuropathy Impairment Score; *NT-proBNP N*-terminal pro-brain-type natriuretic peptide; *PND* polyneuropathy disability; *RBP* retinol-binding protein; *TTR* transthyretin

^a^mBMI calculated as kg/m^2^ × albumin (g/L)

^b^Nerve fiber function assessed through nerve conduction studies of 5 attributes (Σ5): quantitative sensory testing by body surface area including touch pressure and heat pain, vibration detection threshold, heart rate response to deep breathing, and postural blood pressure

**Table 3:** Comparison of the neurologic impairment scores used in the evaluation of hATTR amyloidosis

	NIS-LL	NIS	NIS+7	**mNIS+7**	mNIS+7_Ionis_
Total score	88	244	270	**304**	346.3
Assessment (score)					
Motor strength/weakness	Neurologic exam [lower limbs only] (64)	Neurologic exam (192)	Neurologic exam (192)	**Neurologic exam (192)**	Neurologic exam (192)
Reflexes	Neurologic exam [lower limbs only] (8)	Neurologic exam (20)	Neurologic exam (20)	**Neurologic exam (20)**	Neurologic exam (20)
Sensation	−	−	−	**QST – heat pain and touch pressure at multiple sites (80)**	QST – heat pain and touch pressure at multiple sites (80)
	Neurologic exam [lower limbs only] (16)	Neurologic exam (32)	Neurologic exam (32)	−	Neurologic exam (32)
	−	−	Vibration detection threshold (3.7)	−	−
Composite nerve conduction score	−	−	Σ5 – sural SNAP/ fibular nerve CMAP, tibial motor nerve distal latency, motor nerve conduction velocity, motor nerve distal latency (18.6)^a^	**Σ5 – ulnar CMAP and SNAP, peroneal** ^**c**^ **CMAP, tibial CMAP, sural SNAP (10)** ^**b**^	Σ5 – ulnar CMAP and SNAP, peroneal^c^ CMAP, tibial CMAP, sural SNAP (18.6)^a^
Autonomic function	−	−	Heart rate response to deep breathing (3.7)^a^	**Postural blood pressure (2)** ^**b**^	Heart rate response to deep breathing (3.7)^a^

*CMAP* compound muscle action potential; *exam* examination; *mNIS+7* modified NIS+7; *NIS* Neuropathy Impairment Score; *NIS-LL* NIS based on examination of lower limbs only; *QST* quantitative sensory testing; *SNAP* sensory nerve action potential

^a^Score expressed as normal deviates (0–3.72) based on healthy-subject parameters

^b^Score graded according to defined categories: normal (95th percentile) = 0 points; mildly reduced (≥95th to <99th percentile) = 1 point; and very reduced (≥99th percentile) = 2 points

^c^May also be referred to as fibular [62]

**Fig. 2:**
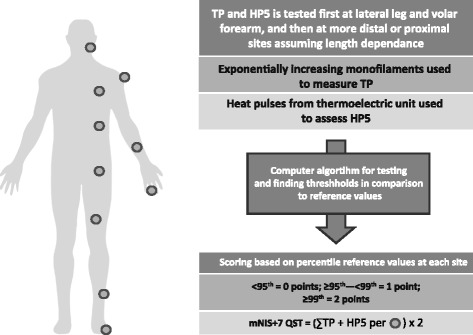
Schematic representation of smart QSTing of body sites. Smart testing uses defined and quantitated stimuli of touch pressure and heat as pain using validated computer software, standard conditions, and previously obtained reference values from the assessment of healthy-subject cohorts. Dots indicate testing site. *HP5*, heat as pain 5 (graded 1–10); *mNIS+7* modified NIS+7; *NIS* Neuropathy Impairment Score; *S ST QSTing* Smart Somatotopic Quantitative Sensory Testing; *TP* touch pressure [17, 62]

The mNIS+7 assessment tool [17] used in this study is a 304-point composite measure of neurologic impairment that includes: neurologic examination of lower limbs, upper limbs, and cranial nerves (NIS-weakness and reflexes); electrophysiologic measures of small and large nerve fiber function (including nerve conduction studies [NCS] Σ5 of ulnar, peroneal, and tibial compound muscle action potential [CMAP] amplitudes and sural and ulnar sensory nerve action potential [SNAP] amplitudes; and smart somatotopic quantitative sensory testing [S ST QSTing; including touch pressure and heat pain]) at defined body surface locations (Fig. 2); and autonomic function (postural hypotension). Scoring for NCS and postural hypotension is based on grading of function: normal (<95th percentile) = 0 points; mildly reduced (≥95th to <99th percentile) = 1 point; and very reduced (≥99th percentile) = 2 points. The mNIS+7 is measured in replicates during the pre-randomization phase (at screening/baseline [21 days before the first dose of study drug] and at baseline [≥24 h after screening/baseline measure]), and at 9 and 18 months. Two independent assessments are taken at each time point, performed at least 24 h apart, but no greater than 7 days apart. An increase in mNIS+7 score, or in any of its components, indicates worsening impairment.

To standardize the efficacy assessment and minimize variability across the multiple centers, neuromuscular physicians were trained to perform the mNIS+7 evaluation at a central center (Dyck Peripheral Nerve Research Laboratory, Mayo Clinic, Rochester, MN, USA).

#### Secondary and exploratory clinical outcome measures

The secondary and exploratory objectives are to determine the effect of patisiran on a variety of clinical parameters, based on their change from baseline to 18 months (Table 2). Unless stated, parameters are assessed at screening/baseline, baseline, 9, and 18 months.

#### Neurologic and cardiac function

Neurologic function is measured using the NIS+7 tool [48] based on two independent readings as described for mNIS+7. For both mNIS+7 and NIS+7, the individual components of the scores will be reported. Specific motor function assessments include the timed 10-m walk test and hand grip strength test (dynamometer), with measurements performed on separate days. Cardiac function is assessed through echocardiograms and cardiac biomarkers (troponin I and N-terminal pro-brain-type natriuretic peptide).

#### Quality of life/symptoms and health status

QoL assessments include the Norfolk QOL-DN questionnaire [49], a 35-item measure sensitive to small fiber, large fiber, and autonomic nerve function, which has been shown to be a reliable indicator of disease severity in patients with hATTR amyloidosis [20] (increase in score = worsening QoL), and the EQ-5D™ questionnaire [50] (decrease in score = worsening QoL). Autonomic symptoms are evaluated using the 31-question Composite Autonomic Symptom Score (COMPASS)-31 questionnaire, which covers six autonomic domains (orthostatic intolerance, vasomotor, secretomotor, gastrointestinal, bladder, and pupillomotor) [51] (increase in score = worsening symptoms). Activity and social function are assessed through the Rasch-built Overall Disability Scale (R-ODS), a 24-item scale to capture limitations on everyday activities [52] (decrease in score = worsening disability). Nutritional status is gauged using mBMI (kg/m^2^ × albumin [g/L]), taken at baseline, and days 84, 189, 357, 462, and 546 (18-month assessment). Changes in ambulation are assessed according to FAP stage [7] and PND score [53] (increase in stage/score = worsening impairment), and the timed 10-m walk test (increase in duration = worsening impairment). Lower limb nerve injury is serially evaluated via voluntary magnetic resonance neurography approximately every 6 months in patients providing voluntary consent.

#### Pathologic nerve fiber and amyloid evaluation

Nerve fibers and amyloid deposits in the skin are quantified through measurement of intraepidermal nerve fiber density, sweat gland nerve fiber density, and dermal amyloid burden using tandem 3 mm skin punch biopsies in patients providing voluntary consent. At each time point, one set of biopsies is taken from the lower leg and another from the distal thigh.

### Pharmacodynamic assessments

Levels of serum TTR protein, vitamin A, and retinol-binding protein (RBP) are measured at baseline, pre-dose on day 0, and on days 21, 126, 252, 253–272 (9 months), 273, 399, 546, and 553–560 (18 months). Serum TTR is measured using an enzyme-linked immunosorbent assay and a turbidimetric assay. Serum RBP is quantified using nephelometry. Serum samples are evaluated by a high-performance liquid chromatography assay to determine vitamin A levels. Pharmacodynamic samples are not taken at 18 months for patients who discontinue treatment at 9 months (modified schedule).

### Pharmacokinetic assessments

Plasma samples for pharmacokinetic analysis are taken pre-dose and post-dose on days 0, 21, 126, 252, 399, and 546. Pharmacokinetic parameters for plasma siRNA are evaluated using a validated ATTO™-probe high-performance liquid chromatography assay.

### Safety assessments

Adverse events (AEs) are assessed throughout the study, and reported according to the Medical Dictionary of Regulatory Activities (version 18.0 or later). AEs are graded based on their severity (mild, moderate, or severe) and the causal relationship to study drug or premedication recorded. Clinical laboratory and chemistry tests, thyroid function parameters, urinalysis, anti-drug antibodies, electrocardiograms, physical and vital signs, and ophthalmology examinations (including electroretinography) are also monitored. The Columbia-Suicide Severity Rating Scale (C-SSRS) is used to assess mental status as it relates to suicidal ideation and behavior.

### Statistical analyses

A sample size of approximately 200 patients will provide 90% power to test a treatment difference of 8.95 points (37.5%) in mNIS+7 change from baseline (primary endpoint) with a two-sided α = 0.05. The sample size of approximately 200 is based on an assumed premature discontinuation rate of 25%.

The populations analyzed in the study are: the modified intent-to-treat (mITT) population (all patients who were randomized and received at least one dose of study drug); the per-protocol population (all patients who completed all efficacy assessments and did not have any major protocol violations); and the safety population (all patients who received at least one dose of study drug).

The analysis of efficacy endpoints will be conducted for the mITT population. Formal statistical hypothesis testing will be performed on the primary and secondary efficacy endpoints with all tests conducted at the nominal two-sided, 0.05 level of significance.

## Discussion

The APOLLO study examines the efficacy and safety of the investigational RNAi therapeutic patisiran in a broad hATTR amyloidosis population, including patients with any amyloidogenic *TTR* mutation and with a wide range of neuropathy severity and age at baseline. The composite primary endpoint of APOLLO (mNIS+7) is well suited for use in hATTR amyloidosis because it encompasses multiple aspects of the polyneuropathy and can capture changes during disease progression. In addition, QoL, motor function, health status, autonomic symptoms, cardiac assessments, and everyday functioning are included as secondary or exploratory endpoints to assess the impact of patisiran on a range of disease involvement.

The mNIS+7 used to assess neurologic impairment in this study is more robust and comprehensive than the tools used in the trials of tafamidis (NIS-LL) [19] or diflunisal (NIS+7) [24], with the modifications outlined in Table 3. One of the key attributes of the mNIS+7 versus NIS+7 is the measurement of sensation, with S ST QSTing used in preference to NIS-sensation evaluation and vibration detection threshold. Indeed, the NIS-sensation score is not included in mNIS+7 in APOLLO, as the study of Suanprasert et al. in 97 patients with hATTR amyloidosis suggested that this score did not adequately capture sensation loss [17]. Compared with NIS-sensation, S ST QSTing provides an improved balance between large and small sensory nerve fibers and measures sensation loss over the whole body rather than at distal sites such as big toes and fingers. Whilst S ST QSTing is time-consuming and requires specialist training and standardized protocols to avoid procedural variability [54, 55], these demands are necessary to assess sensation loss somatopically and accurately [56]. Aside from sensation, the other major alteration from the NIS+7 is in the measurement of nerve conduction. Here, the combined NCSΣ5 in the mNIS+7 includes only action potential amplitudes, which are more suited to capture changes in disease course for hATTR amyloidosis as this disease has a primarily axonal pathophysiology. A further change from the NIS+7 is the new autonomic measure: postural hypotension was included because heart rate decrease with deep breathing was considered an inconsistent assessment of autonomic function as it is unevaluable in patients with cardiac pacemakers and frequently unevaluable in patients with arrhythmias [17, 56]. It is recognized however, that pharmacologic interventions (e.g., fludrocortisone and midodrine) can be used to treat postural hypotension [57], and the use of any such strategies should be considered when gauging the effect of patisiran on this measure. With the modifications to the NIS+7, the total score was increased to 304 points for the mNIS+7 used in this study. Measurement of muscle weakness and stretch reflexes were considered adequate with the original NIS+7, so these elements have not been modified [17].

However, it should be noted that the mNIS+7 described by Suanprasert et al., which is used in APOLLO, is different from that currently being used in the study investigating the anti-TTR antisense oligonucleotide IONIS-TTR_Rx_ (mNIS+7_Ionis_). The critical difference between these two versions of the mNIS+7 is in the measurement of sensation: mNIS+7_Ionis_ includes the NIS-sensation testing score from the original NIS+7, in addition to S ST QSTing [56] (Table 3). As discussed, NIS-sensation may not provide a true reflection of sensation impairment, with inclusion of the original NIS+7-sensation testing potentially being redundant. Indeed, this may lead to double counting for assessments of sensation. The other notable difference is that the mNIS+7_Ionis_ does not include postural hypotension, and instead retains the heart rate decrease with deep breathing measure of the NIS+7 for assessment of autonomic function, which has the limitations discussed above for patients with pacemakers or cardiac arrhythmias. Both mNIS+7 and mNIS+7_Ionis_ include only CMAPs and SNAPs as nerve conduction measures (Table 3). Regarding assessment of NCS, it is worth noting that these are expressed as normal deviates in the mNIS+7_Ionis_, whereas they are graded by defined categories in mNIS+7 (Table 3).

The features of the mNIS+7 described above have been introduced to create a tool suitable for use in hATTR amyloidosis clinical studies. As assessment of neuropathic measures is subject to variability between investigators, extensive training is provided to support the use of a standardized and validated methodology and ensure that scoring is consistent and accurate. Specifically, specialized training is provided for neuromuscular physicians, with certification upon completion. For clinical assessment of neuropathy signs and symptoms, neuromuscular experts are trained to use only unequivocal abnormalities (accounting for age, sex, physical fitness, and anthropomorphic variables) rather than more traditional clinical criteria, and not to grade for concomitant neuromuscular disease. Previous analyses have shown that this strategy, when used by trained specialists, leads to increased reproducibility and a notable improvement in proficiency when scoring clinical measures, such as weakness and reflexes, as used in APOLLO [58, 59]. Variability between centers has also been noted for NCS, which is countered in this study through specialist training on techniques and reference values, and evaluation of tracings at a central reading center; these methodologies have been shown to reduce inter-investigator variability [60, 61]. Of the other mNIS+7 measures, the S ST QSTings used in APOLLO are also standardized and referenced, to ensure generation of accurate and comparable data across the study [62].

The clinical relevance of NIS has been previously demonstrated, with total NIS correlated with FAP stage, PND score, and QoL (Norfolk QOL-DN and SF-36) in patients with hATTR amyloidosis [47, 56]. In addition, rapid worsening of NIS was observed in untreated patients in a natural history study of patients with hATTR amyloidosis [47], fitting with the relentless progression expected for this disease [8]. The estimated rate of NIS progression for a patient with a baseline NIS of 32 was 14.3 points per year [47], and a separate analysis has indicated a worsening in NIS-LL of up to 7.4 points over 12 months in patients with a baseline NIS-LL of 20–30 [63]. The rapid neurologic deterioration observed in patients with hATTR amyloidosis contrasts with lower rates of NIS progression seen in other neuropathies [64, 65]. For example, in patients with mild-to-moderate diabetic distal symmetric sensorimotor polyneuropathy, NIS and NIS-LL increased by only 0.61 and 0.43 points, respectively, over 4 years of placebo treatment [64]. Furthermore, a study of disease progression in Charcot–Marie–Tooth disease type 1A reported an annual NIS increase of 1.37 points [65].

**Fig. 3:**
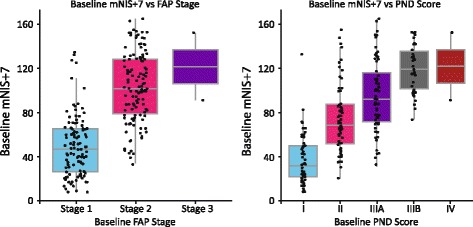
Association between mNIS+7 and (A) FAP stage and (B) PND score using baseline data from the APOLLO study (*n* = 225). *FAP* familial amyloidotic polyneuropathy; *mNIS+7* modified Neuropathy Impairment Score +7; *PND* polyneuropathy disability

Based on the described modifications to the NIS, it is anticipated that the mNIS+7 will more accurately capture the degree of polyneuropathy and neurologic impairment in patients with hATTR amyloidosis. Indeed, rapid disease progression in untreated patients has also been demonstrated using the mNIS+7, with an increase of approximately 24 points anticipated after 18 months [47]. The clinical value of the mNIS+7 was further demonstrated in recent analyses of the baseline data from APOLLO, which showed an association between mNIS+7 and FAP stage and PND score (Fig. 3). In addition, investigation of the mNIS+7_Ionis_ showed that clinical polyneuropathy signs and symptoms correlated strongly with assessments of action potential amplitudes and somatotopic touch pressure [56], which are also included in the mNIS+7. Of the other measures taken in APOLLO, a correlation between mNIS+7 and TTR knockdown was demonstrated in the Phase 2 open-label extension study of patisiran [66], supporting the hypothesis that reducing levels of amyloidogenic protein leads to clinical benefit in hATTR amyloidosis (e.g., via OLT [28]).

hATTR amyloidosis is associated with symptoms across multiple systems [2, 67]. In addition to the neurologic symptoms, patients often present with a mixed phenotype including concurrent cardiac symptoms. Cardiomyopathy associated with hATTR amyloidosis can lead to heart failure and death, highlighting the need for vigilance around cardiac amyloid fibril accumulation. The APOLLO study therefore includes echocardiographic and biochemical cardiac parameters to assess the impact of patisiran on cardiac progression.

The impact of the diverse disease symptoms may not be fully captured by clinical and laboratory examinations, so QoL measures were included as key endpoints in the studies of tafamidis and diflunisal [19, 24]. APOLLO uses the Norfolk QOL-DN and EQ-5D questionnaires to assess QoL. The Norfolk QOL-DN questionnaire evaluates small and large nerve fiber function in addition to autonomic impairment and activities of daily living. It has demonstrated utility in patients with hATTR amyloidosis with polyneuropathy both as a measure of disease severity and as a clinical endpoint to assess response to treatment [20, 21]. The EQ-5D questionnaire has been used as part of the international Transthyretin Amyloidosis Outcomes Survey (THAOS), which demonstrated worsening QoL with disease progression [22]. Autonomic symptoms, particularly gastrointestinal events, are common in patients with hATTR amyloidosis [23] underpinning the comprehensive assessment of autonomic function using the COMPASS-31 questionnaire [51]. Other measures in APOLLO include the R-ODS survey [52] for assessment of the effects of patisiran on activities of daily living. The R-ODS has already been used to demonstrate that hATTR amyloidosis can affect activities such as washing dishes or fastening buttons [68], and has been validated in patients with V30M hATTR amyloidosis [69].

The pharmacotherapies currently approved or available for hATTR amyloidosis can slow or sometimes stabilize disease, but there is little evidence that complete stabilization or reversal of nerve damage can be achieved. Sensory and autonomic innervation in skin punch biopsies are being evaluated in APOLLO to determine whether patisiran can increase nerve fiber density. Increases in sweat gland fiber density were observed in the Phase 2 open-label extension study of patisiran [46], and APOLLO will provide an opportunity for these findings to be validated in a placebo-controlled setting. Of interest, sweat gland nerve fiber density has previously been associated with ambulation in patients with hATTR amyloidosis [70]. In addition to nerve fiber density, these skin biopsies are also being evaluated in both the Phase 2 open-label extension study and APOLLO for changes in dermal amyloid burden.

Aside from clinical endpoints related to efficacy, safety and pharmacodynamics will also be assessed throughout the APOLLO study and subsequent open-label extension. Longer-term data from the Phase 2 open-label extension study suggest patisiran is well tolerated, but APOLLO will allow comparison against a placebo-controlled arm. In particular, the safety of prolonged lowering of vitamin A levels, associated with TTR reduction, will be assessed. TTR reduction itself provides a convenient biomarker to assess patisiran activity, and reduction in the pathogenic protein may also relate to clinical parameters.

Recruitment for APOLLO started in December 2013 and was completed in January 2016, with 225 patients enrolled. This trial represents the largest Phase 3 study of an RNAi strategy for the treatment of hATTR amyloidosis, with clinical endpoints that strive to assess the multiple ways in which this disease affects patient well-being and function. The Patisiran Global open-label extension study was initiated in July 2015, and will provide further data on long-term safety and efficacy of patisiran in patients with hATTR amyloidosis.

## Abbreviations

AE: Adverse event
CMAP: Compound muscle action potential
COMPASS-31: 31-question Composite Autonomic Symptom Score
EQ-5D: EuroQol 5-Dimensions
FAP: Familial amyloid polyneuropathy
hATTR amyloidosis: Hereditary ATTR amyloidosis
mBMI: Modified body mass index
mITT: Modified intent-to-treat
mNIS+7: Modified Neuropathy Impairment Score
mNIS+7_Ionis_: mNIS+7 being used in the study investigating the anti-TTR antisense oligonucleotide IONIS-TTR_Rx_
NCS: Nerve conduction study
NIS: Neuropathy Impairment Score
NIS-LL: NIS-Lower Limb
OLT: Orthotopic liver transplantation
PND: Polyneuropathy disability score
QoL: Quality of life
QOL-DN: Norfolk Quality of Life-Diabetic Neuropathy
RBP: Retinol-binding protein
RNAi: RNA interference
R-ODS: Rasch-built Overall Disability Scale
SF-36: Short-Form 36
siRNA: Small interfering RNA
SNAP: Sensory nerve action potential
S ST QSTing: Smart somatotopic quantitative sensory testing
TTR: Transthyretin
